# Observational study investigating Ectoin^®^ Rhinitis Nasal Spray as natural treatment option of acute rhinosinusitis compared to treatment with Xylometazoline

**DOI:** 10.1007/s00405-021-06916-0

**Published:** 2021-06-04

**Authors:** Nina Werkhäuser, Andreas Bilstein, Kathrin Mahlstedt, Uwe Sonnemann

**Affiliations:** 1 Bitop AG, Carlo-Schmid-Allee 5, 44263 Dortmund, Germany; 2 AB-MSC, Am Platz 2, 50129 Bergheim, Germany; 3 Reichsstr. 108, 14052 Berlin, Germany; 4 Hermann-Ehlers-Weg 4, 25337 Elmshorn, Germany

**Keywords:** Acute rhinosinusitis, Ectoine, Ectoin^®^ Rhinitis nasal spray, Nasal spray, Xylometazoline

## Abstract

**Introduction:**

Symptomatic relief of acute rhinosinusitis is commonly achieved with nasal decongestants. The current observational study investigated the efficacy and safety of treatment of acute rhinosinusitis with Ectoin^®^ Rhinitis Spray compared to or in combination with Xylometazoline-containing decongesting nasal spray.

**Methods:**

Patients with acute rhinosinusitis applied either Ectoin^®^ Rhinitis Spray, Xylometazoline nasal spray or a combination of both products. Rhinosinusitis symptoms were assessed, and nasal oedema and endonasal redness were determined by rhinoscopy. Patient diaries based on the validated SNOT (Sino Nasal Outcome Test) questionnaire evaluated rhinosinusitis parameters over time and influences of the disease on quality of life. Following treatment, investigators and patients judged the efficacy and tolerability.

**Results:**

Ectoin^®^ Rhinitis Spray diminished common rhinosinusitis symptoms such as nasal obstruction, nasal secretion, facial pain/headache, and smell/taste impairment. Upon treatment over 7 days, rhinosinusitis sum scores decreased statistically significantly (*p* < 0.001) by − 64.25%, which was comparable to that achieved with Xylometazoline-containing decongesting nasal spray (− 67.60%). No side effects were observed during treatment with Ectoin^®^ Rhinitis Spray, whereas treatment with Xylometazoline-containing nasal spray resulted in nasal mucosa dryness. Concomitant treatment with both products diminished the development of nasal dryness and required fewer applications of Xylometazoline-containing nasal spray.

**Conclusion:**

Ectoin^®^ Rhinitis Spray is an effective, natural treatment option for acute rhinosinusitis, which may be used as monotherapy or as add-on treatment with a Xylometazoline-containing nasal spray. The concomitant use of Ectoin^®^ Rhinitis Spray might reduce the needed dose of decongestant nasal spray and counteract bothersome side effects such as dry nasal mucosa.

**Trial registration:**

The current study was registered in the ClinicalTrials.gov database under the identifier: NCT03693976 (date of registration: Oct 3, 2018).

## Introduction

Rhinosinusitis is a very prevalent condition with significant negative impact on patient’s quality of life (QoL) and socioeconomic burden [1, 2]. According to the European Position Paper on Rhinosinusitis and Nasal Polyps (EPOS 2012), rhinosinusitis is defined as inflammation of the nose and the paranasal sinuses characterised by two or more symptoms, one of which should be nasal obstruction or nasal discharge (anterior/posterior nasal drip) and which may be accompanied by facial pain/pressure, impairment of taste and smell, and cough [3]. Depending on the course of the disease, rhinosinusitis is classified as acute (symptoms/medical condition present for ≤ 4 weeks), subacute (symptoms/medical condition present between 4 and 12 weeks) recurrent (at least 4 episodes of rhinosinusitis within 12 months) or chronic (symptoms/medical condition present for > 12 weeks) form [1]. Acute rhinosinusitis is further categorized as bacterial, viral (common cold) or post-viral [3].

Several treatment recommendations exist for rhinosinusitis, and a stepwise approach should be followed starting with symptomatic relief strategies such as decongestants [3, 4]. Decongestant nasal sprays with Xylometazoline have been administered to treat acute rhinosinusitis for more than 30 years. However, due to the risk of habituation effects or chronic nasal congestion, their use is limited to a period of about one week and often results in dryness of the nasal mucosa [5, 6].

The current study investigated the efficacy and safety of Ectoin^®^ Rhinitis Nasal Spray and evaluated its concomitant use with a Xylometazoline-containing nasal spray. Ectoine is a low molecular weight molecule, which was originally discovered as new amino acid derivate in a bacterium of the genus *Ectothiorhodospira *[7]. It is classified as a compatible solute allowing bacteria to counteract negative effects of high osmolarity or other extreme environments [8, 9]. Ectoine works via a mechanism called “preferential exclusion”, resulting in the preferential hydration of macromolecules, thereby protecting those from negative influences [10]. The industrial-scale production of ectoine has provided the opportunity to employ its protective and moisturizing effects into a range of medical devices. Their successful application as, e.g., nasal sprays, throat sprays, lozenges or inhalation solutions has been demonstrated in a number of clinical trials [11]. Whereas most of the former studies demonstrated the application of Ectoin^®^ containing nasal spray in allergic rhinitis [12], the current study served to explore the application of an Ectoin^®^ containing nasal spray in treating acute viral rhinosinusitis. In a prior conducted clinical trial, it had been demonstrated that acute rhinitis can be treated comparably well with Ectoin^®^ Rhinitis Spray as with a systemic treatment using herbal tablets [13]. The current study aimed to compare its efficacy with commonly applied decongesting nasal sprays. It was hypothesised that treatment with Ectoin^®^ Rhinitis Spray may counteract the dehydration of the nasal mucosa which is commonly observed when applying Xylometazoline, and it was aimed to study if concomitant treatment may reduce the used dose of Xylometazoline.

## Materials and methods

### Study design

The current study was a multi-centre, observational study, which was carried out with incoming patients in two ear nose throat doctor’s offices in Germany.

### Study patients and treatments

Patients aged ≥ 6 years diagnosed with acute rhinosinusitis were asked for their participation (V1/day 0). After signing an informed consent, patients freely choose to apply the CE-registered medical device (Ectoin^®^ Rhinitis Nasal Spray [ectoine] containing Ectoin^®^ med (a specific ectoine manufactured by bitop AG, Dortmund, Germany), sodium chloride, sodium-di-hydrogen-phosphate dihydrate, di-sodium-hydrogen-phosphate, and water) or the drug (0.1% Xylometazoline-containing nasal spray (NasenSpray-ratiopharm^®^ Erwachsene) [Xylo], Ratiopharm, Ulm, Germany) or a combination of both products [ectoine + Xylo]. Both products were applied preservative free with the same pump system (3 K, Ursatec). Products were applied within their defined intended use and in accordance with their respective instructions for use. Patients were asked to return to the study site on day 7 ± 2 for a regular control visit (V2).

### Study assessments

During the initiation visit (V1/day 0), eligibility criteria (presence of rhinosinusitis sum score of ≥ 8, absence of acute bacterial or chronic rhinosinusitis) were checked. Rhinosinusitis symptoms (nasal obstruction, nasal secretion, facial pain/headache, and smell/taste disorders) were assessed at V1 and at V2 based on a 5-point scale ranging from 0 = no symptoms to 4 = very strong symptoms. The sum score was calculated as the sum of all single rhinosinusitis symptoms. In addition, dryness of the nasal mucosa, severity of sore throat and the level of endonasal oedema and redness assessed by rhinoscopy (assessed by endoscopy) were evaluated at V1 and V2, using the same rating scales as for the rhinosinusitis symptoms. The general wellbeing of patients was determined on a 4-point scale ranging from 0 = good condition to 3 = bad condition at V1 and V2.

Patients were asked to document their disease-specific health status in a patient diary for the entire study duration (day 0 to day 7 ± 2). The patient diary was based on the validated SNOT-22 (Sino-Nasal-Outcome-Test) questionnaire [14, 15], which was extended with the parameter “dry nose”. Assessment of symptoms was based on a 6-point scale ranging from 0 = no problem to 5 = as bad as possible. In addition, patients were asked to indicate the 5 items which were most bothersome to them.

At the end of the study, investigators and patients were asked to rate the overall efficacy and tolerability of treatments on a 6-digit scale with 1 = very good until 6 = very bad.

### Study endpoints

The main objective of the current study was to evaluate the efficacy and tolerability of the Ectoin^®^ Rhinitis Nasal Spray in routine clinical practice in patients with acute viral rhinosinusitis in comparison to treatment with a Xylometazoline-containing nasal spray. The primary outcome measure was the physician’s assessment of the change of intensity of rhinosinusitis symptoms over time. Secondary outcome measures were the physician’s assessment of general well-being of patients, patient’s assessment of intensity of symptoms and their influence on quality of life, investigator’s and patient’s assessment of efficacy and tolerability of treatments, and incidence of adverse events.

### Power calculation and statistical analysis

The power calculation of the current study was based on results of a former clinical study, which was carried out in patients with acute rhinosinusitis treated either with Xylometazoline nasal spray or concomitantly with Xylometazoline nasal spray and an Ectoin^®^ containing Nasal Douche (results not yet published). Based on the reached improvement of acute rhinosinusitis sum scores in the former trial, the power calculation showed that 51 completed patients per group would be necessary to reach 80% power. Taking into account the potential dropout of patients, it was decided to aim for 56 participating patients per group. The statistical analysis was carried out using SAS^®^, Version 9.3 (SAS Institute Inc., Cary, NC, USA). Continuous values were described using mean, standard deviation, range (minimum, maximum) and number of valid results. 95% confidence intervals (CI) were added, if deemed useful. Categorical data were described using absolute frequencies and percentages. Study end points were described descriptively and over the course of time. Intra-individual differences between time points were analysed for significance using a Wilcoxon-rank test, and differences between groups were analysed for significance using a Wilcoxon sum test. Evaluations of safety and tolerability were analysed descriptively considering absolute numbers, frequency, type, severity grade and relationship to study treatments. Graphical images were prepared with SAS^®^, Version 9.3 (SAS Institute Inc., Cary, NC, USA). and GraphPad Prism (Version 7.05, San Diego, CA, USA).

## Results

From October 2018 until April 2019, 168 patients with acute viral rhinosinusitis were included in the study: 56 patients applied Ectoin^®^ Rhinitis Nasal Spray, 56 patients applied the Ectoin^®^ Rhinitis Nasal Spray and the Xylometazoline-containing nasal spray concomitantly, and 56 patients applied the Xylometazoline-containing nasal spray.

### Baseline characteristics

Demographics of participating patients are listed in Table 1. Patients aged 7 to 84 years were enrolled, and there were no statistically significant differences regarding age, relevant comorbidities, and smoking status between the treatment groups.

**Table 1:** Demographics of participating patients

	Ectoine		Ectoine + Xylo		Xylo	
*n*	56		56		56	
Age, mean [SD]	34.5 [17.28]		40.8 [16.21]		35.4 [16.85]	
Gender, *n* [%]	Female	Male	Female	Male	Female	Male
	39 [69.6]	17 [30.4]	32 [57.1]	24 [42.9]	36 [64.3]	20 [35.7]

*n* number of patients, % percentage based on *n*

### Efficacy

#### Rhinosinusitis symptoms

**Fig. 1:**
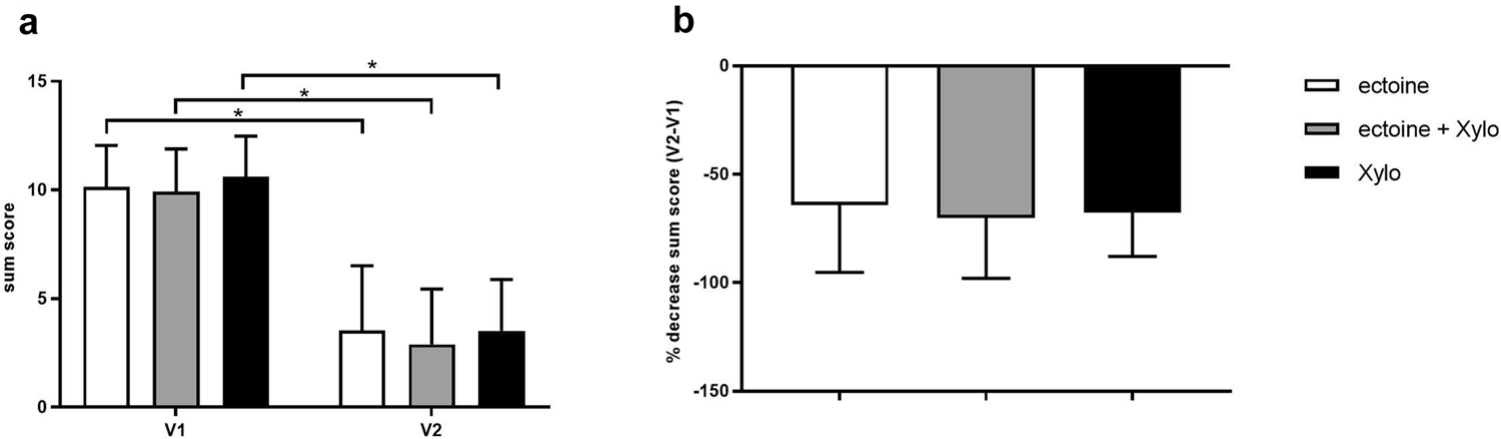
Development of rhinosinusitis sum scores in patients treated with Ectoin^®^ Rhinitis Spray (ectoine), Ectoin^®^ Rhinitis Spray and Xylometazoline spray (ectoine + Xylo) or Xylometazoline spray (Xylo). **a** Total sum scores (mean ± SD) assessed at visit 1 (V1) and at visit 2 (V2). *p < 0.0001. **b** Percentual decrease of sum scores (mean ± SD) from visit 1 (V1) to visit 2 (V2)

During the initiation visit (V1/day 0), patients showed moderate to strong rhinosinusitis symptoms with sum scores (reachable maximum value was 16) of 10.16 in the ectoine group, 9.93 in the ectoine + Xylo group, and 10.61 in the Xylo group. The severity of all single rhinosinusitis symptoms improved statistically significantly from V1 to V2 in all three treatment groups (*p* < 0.0001, see Table 2). This was also reflected in a statistically significant decrease of sum scores in all treatment groups (*p* < 0.0001). The percentual decrease of the severity of the sum score was comparable between groups with a decrease of − 64.25% in the ectoine group, − 70.12% in the ectoine + Xylo group, and − 67.60% in the Xylo group (see Fig. 1).

**Table 2:** Severity of single rhinosinusitis symptoms at the beginning (V1) and at the end (V2) of the study

	Ectoine (mean ± SD)		Ectoine + Xylo (mean ± SD)		Xylo (mean ± SD)	
	V1	V2	V1	V2	V1	V2
Nasal obstruction	2.84 ± 0.78	1.00 0.81	2.96 ± 0.71	0.86 ± 0.84	2.96 ± 0.66	1.00 ± 0.69
Nasal secretion	2.70 ± 0.66	0.98 ± 0.88	2.64 ± 0.80	0.75 ± 0.69	2.86 ± 0.67	0.91 ± 0.61
Facial pain and headache	2.34 ± 0.92	0.91 ± 1.05	2.18 ± 0.96	0.70 ± 0.85	2.38 ± 1.00	0.89 ± 1.02
Smell/taste disorders	2.29 ± 0.85	0.68 ± 0.81	2.14 ± 1.02	0.59 ± 0.85	2.41 ± 0.99	0.71 ± 0.82
Sum score	10.16 ± 1.90	3.55 ± 2.97	9.93 ± 1.96	2.89 ± 2.55	10.61 ± 1.87	3.52 ± 2.36

Symptoms were evaluated on a 5-point scale from *0*  none, *1*  light, *2* moderate, *3*  strong to *4*  very strong symptoms. Reduction of all symptoms was significant (*p *< 0.05)

#### Nasal dryness and sore throat

**Fig. 2:**
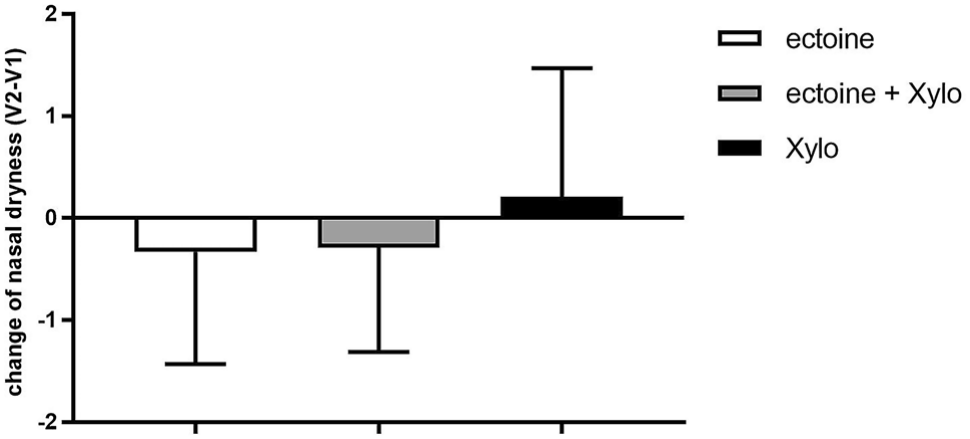
Change in nasal dryness from V1 to V2 (mean ± SD). Ectoine = Ectoin^®^ Rhinitis Nasal Spray, Xylo = Xylometazoline nasal spray. Dryness was assessed on a scale from 0 = none to 3 = very strong

The symptoms nasal dryness and sore throat were comparably (and statistically not differently) pronounced in all three treatment groups at V1: mean values of “dryness of nasal mucosa” were 0.68 ± 0.0.94 (ectoine), 0.91 ± 1.03 (ectoine + Xylo) and 0.95 ± 1.03 (Xylo). As shown in Fig. 2, treatment with Xylometazoline nasal spray resulted in increased dryness of the nasal mucosa (1.16 ± 0.95). In contrast, treatment with the Ectoin^®^ Rhinitis Spray improved the nasal dryness statistically significantly (0.36 ± 0.55, *p* = 0.034), as did treatment with the combination of both products (0.63 ± 0.82, *p* = 0.047).

Mean values of the symptom sore throat were 0.86 ± 0.1.0 (ectoine), 1.18 ± 0.94 (ectoine + Xylo) and 1.02 ± 0.0.9 (Xylo) at V1, which clearly improved in all three treatment groups without differences between groups.

#### Nasal oedema, endonasal redness, and general well-being

**Fig. 3:**
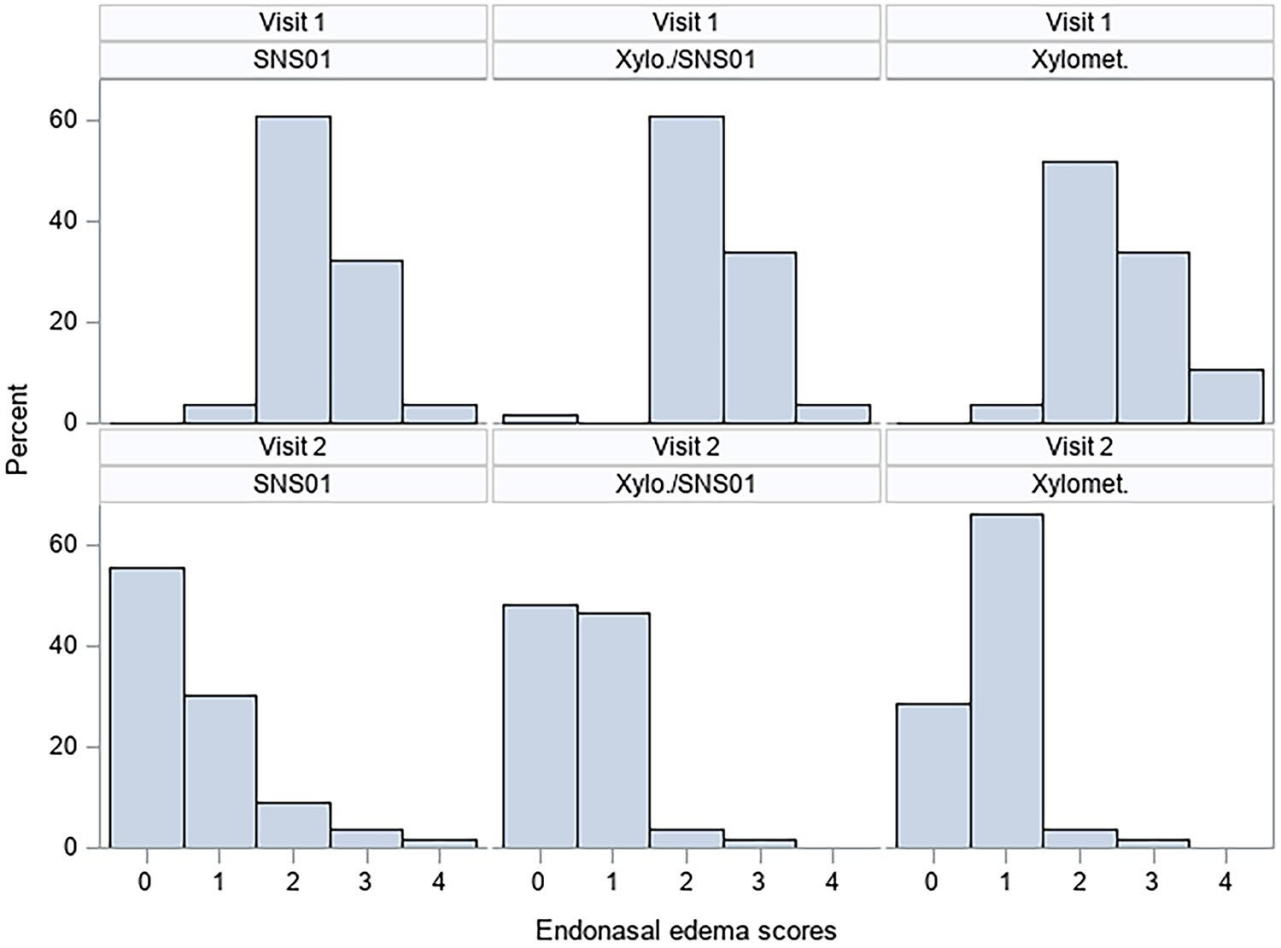
Distribution (% of patients) of endonasal oedema severity scores (0 = no symptoms, 4 = very strong symptoms) at V1 and V2: patients were either treated with Ectoin^®^ Rhinitis Nasal Spray (SNS01), Ectoin^®^ Rhinitis Nasal Spray + Xylometazoline nasal spray (Xylo/SNS01) or Xylometazoline nasal spray (Xylomet)

The rhinoscopic determination of endonasal oedema and redness demonstrated that the distribution of the severity scores of endonasal oedema differed significantly at V2 comparing patients treated with Ectoin^®^ Rhinitis Spray versus patients treated with Xylometazoline (*p* = 0.0011). Similarly, the distribution of the severity scores of endonasal redness differed significantly at V2 comparing the treatment groups ectoine versus Xylo (*p* < 0.0001) and comparing the treatment groups ectoine + Xylo versus Xylo (*p* = 0.0448). Differences of severity scores of endonasal oedema are depicted in Fig. 3. Although the distribution of severity grades of the symptoms differed significantly between the groups, mean changes of rhinoscopy values over time (V2-V1) did not differ statistically significantly.

The general wellbeing of patients improved statistically significantly in all three treatment groups (*p* < 0.0001 for all groups) from V1 (mean values ectoine: 2.18 ± 0.54, ectoine + Xylo: 2.23 ± 0.57, Xylo: 2.25 ± 0.51) to V2 (mean values ectoine: 0.55 ± 0.78, ectoine + Xylo: 0.64 ± 0.8, Xylo: 0.66 ± 0.64) without differences between groups.

#### Patient’s assessment of rhinosinusitis symptoms

Patients were asked to document their symptoms in a patient diary which was based on the validated SNOT-20 questionnaire [14]. Questions of the diary covered the judgement of typical rhinosinusitis symptoms as well as patient’s quality of life.

Severity of rhinosinusitis symptoms was assessed by judging i.e., need to blow nose, sneezing, runny nose, nasal congestion, dry nose, impairments in smell/taste, cough, postnasal discharge, thick nasal discharge, ear fullness, dizziness, ear pain, and facial pain/pressure.

Upon treatment (from day 0 to day 7), all rhinosinusitis parameters clearly improved in all three treatment groups. Independent on treatment, patients assessed the parameters of need to blow the nose, runny nose, and nasal congestion as three out of five most bothersome rhinosinusitis symptoms. When comparing single-day values, some differences were found regarding actual values on single days; however, overall development of parameters was comparable between groups.

**Fig. 4:**
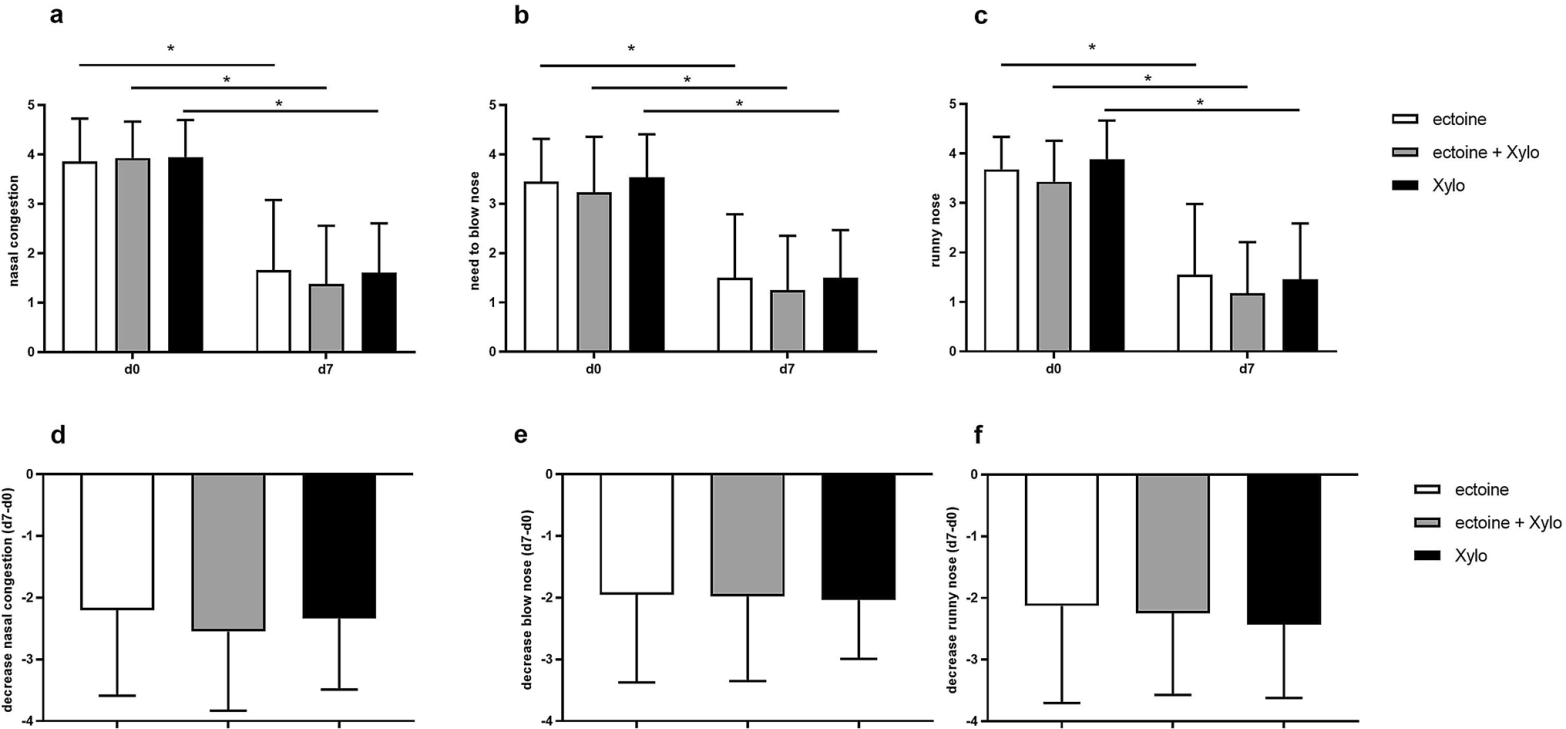
Development of the most bothersome symptoms nasal congestion (**a**), the need to blow the nose (**b**), and runny nose (**c**) depicted as total values on day 0 (d0) and day 7 (mean ± SD). Respective percentual decrease (mean ± SD) from day 0 to day 7 is shown in **d**–**f**. Patients evaluated symptoms on a scale ranging from 0 = no problem to 5 = as bad as possible. *p < 0.0001

As depicted in Fig. 4, all three most bothersome symptoms decreased significantly (*p* < 0.0001) from day 0 to day 7 without significant differences between groups.

Comparing changes of values over the entire study duration (d7-d0) indicated that actual values did not differ between groups except for the symptom cough, which was significantly greater improved in the combination group (− 1.57 ± 1.70) compared to the group using Ectoin^®^ Rhinitis Nasal Spray (− 0.96 ± 1.73; *p* = 0.041).

When comparing the percentual changes (day 7–day 0) of rhinosinusitis symptoms, significant differences between groups were determined for the following parameters:

The symptom “dry nose” decreased by − 54.67 ± 70.18% from day 0 until day 7 in the Ectoin^®^ Rhinitis Nasal Spray group, whereas only a decrease of − 37.52 ± 49.49% from day 0 to day 7 was determined in the group applying Xylometazoline (*p* = 0.0164).

The percentual decrease of the symptom ear pain was − 87.22 ± 28.60% in the Ectoin^®^ Rhinitis Nasal Spray group, whereas the decrease was significantly less pronounced in the group using both Ectoin^®^ Rhinitis Nasal Spray + Xylometazoline, with a percentual decrease of − 61.62 ± 86.42% (*p* = 0.0309).

#### Patient’s assessment of other physical and emotional parameters

In addition to the assessment of typical rhinosinusitis symptoms, patients evaluated physical problems (e.g., difficulty falling asleep, waking up at night, lack of good sleep, wake up tired), functional limitations (e.g., fatigue, reduced productivity, reduced concentration), and emotional consequences of rhinosinusitis (e.g., being frustrated, sad, embarrassed).

Overall, all assessed parameters improved statistically significantly in all treatment groups from day 0 until day 7. Comparing treatments, a greater improvement of the parameter “difficulties falling asleep” was observed in the group using Xylometazoline (− 2.29 ± 1.52) compared to the group using Ectoin^®^ Rhinitis Nasal Spray (− 1.70 ± 1.40, *p* = 0.032). Similarly, the symptom “fatigue” improved significantly better in the Xylometazoline group (*p* = 0.0334), where decreases of − 75.90 ± 26.92% were determined compared to a decrease of − 55.43 ± 47.64% in the Ectoin^®^ Rhinitis Nasal Spray group.

### Efficacy and tolerability

At the end of the study, investigators and patients were asked to rate the overall efficacy and tolerability of the treatments (on a 6-point scale from 1 = very good to 6 = insufficient). The efficacy of Xylometazoline was judged as good by investigators (1.86 ± 0.52 for the ectoine + Xylo group, 1.89 ± 0.85 for the Xylo group) and by patients (1.79 ± 0.56 for the ectoine + Xylo group, 1.84 ± 0.83 for the Xylo group), and there were no statistical differences between the groups. Similarly, judgment of the efficacy of the Ectoin^®^ Rhinitis Spray reflected good scoring without significant differences between the group treated with Ectoin^®^ Rhinitis Spray and the group treated with the combination of both products (investigators: 2.48 ± 1.26 for the ectoine group, 2.46 ± 0.99 for the ectoine + Xylo group; patients. 2.50 ± 1.32 for the ectoine group, 2.39 ± 1.04 for the ectoine + Xylo group). Overall, investigators and patients judged the efficacy of Xylometazoline treatment (both as monotherapy and as combination therapy) significantly better (*p* < 0.0001) than that of the Ectoin^®^ Rhinitis Spray.

Importantly, the investigator’s assessment demonstrated that the tolerability of the Ectoin^®^ Rhinitis Spray was evaluated significantly better (1.54 ± 0.69 for the ectoine group, 1.64 ± 0.72 in the ectoine + Xylo group) than the tolerability of Xylometazoline nasal spray (1.89 ± 0.85 for the ectoine + Xylo group, 2.07 ± 0.95 for the Xylo group; *p* = 0.0004). This was confirmed by the patient’s judgement, assessing the tolerability of Ectoin^®^ Rhinitis Spray as significantly better (1.52 ± 0.74 in the ectoine group, 1.64 ± 0.72 in the ectoine + Xylo group) than that of Xylometazoline (1.86 ± 0.86 for the ectoine + Xylo group, 2.09 ± 1.01 for the Xylo group; *p* = 0.0008).

### Dosage of treatments

Overall (considering the doses of each product from groups applying either one product or the combination of two products), the Ectoin^®^ Rhinitis Spray dose decreased from a mean of 11.5 ± 3.12 sprays per day on day 1 to a mean of 7.9 ± 4.95 sprays per day on day 7. The mean Xylometazoline dose per day (including both the group using Xylometazoline and the group using a combination of the two study products) decreased from 7.9 ± 3.00 sprays per day on day 1 to 4.8 ± 2.47 sprays per day on day 7.

The dose of Xylometazoline nasal spray was slightly lower in the patient group applying the combination of both products (7.4 ± 2.66 on day 1 decreasing to 4.5 ± 2.79 on day 7) compared to the patient group using Xylometazoline alone (8.4 ± 3.25 on day 1 decreasing to 5.0 ± 2.09 on day 7). This difference was statistically significant on day 1 (*p* = 0.0477) and on day 3 (*p* = 0.0218).

### Safety

One adverse event (abscess at the tonsils) was recorded during the study, which was evaluated as not treatment related.

## Discussion

Acute rhinosinusitis is a very common disease with socio-economic burden and negative impact on quality of life. It is commonly treated symptomatically with analgesics, saline irrigation, or decongestants [16–18]. Often, acute rhinosinusitis is self-managed by patients without seeking medical care; therefore, effective, and safe treatment options are essential.

The current study served to investigate the efficacy and tolerability of the Ectoin^®^ Rhinitis Spray, a hypertonic solution containing the natural ingredient ectoine (Ectoin^®^ med, a specific ectoine manufactured by bitop AG, Dortmund, Germany), and to compare it to a Xylometazoline containing nasal spray. In contrast to Xylometazoline, ectoine acts via a physical mode of action [19, 20], therefore representing a non-pharmacological treatment option for acute rhinosinusitis.

In all treatment groups, severity of rhinosinusitis symptoms improved clinically relevant without significant differences between treatment groups. Thus, Ectoin^®^ Rhinitis Nasal spray treatment resulted in comparable improvement of the typical rhinosinusitis symptoms nasal obstruction, nasal secretion, facial pain/headache, and smell/taste disorders as did treatment with a nasal spray containing the pharmacologically acting decongestant Xylometazoline.

Interestingly, the use of nasal decongestants was described as commonly accepted treatment of ARS in the European Position Paper on Rhinosinusitis and Nasal Polyps (EPOS) of the year2012 [3]. The revised version of the EPOS guideline (2020) discusses the use of decongestants, referring to a Cochrane review on the use of decongestants in common cold in adults and children [6] and conclude that decongestants may have a small positive effect on subjective measures of nasal congestion [21]. However, the review included only one study where a topical decongestant was applied (whereas oral decongestants were applied in the remaining studies), and the authors themselves point out that there is a lack of suitable study results, which would allow a general judgement on the use of topical decongestants in common cold. The current study demonstrated that nasal obstruction was statistically reduced upon treatment with the nasal decongestant Xylometazoline and may therefore add valuable data for future evaluation of the general efficacy of topical decongestants.

One commonly occurring side effect of Xylometazoline, the development of dry nasal mucosa, could be counteracted with the concomitant application of Ectoin^®^ Rhinitis Spray. This is of importance as the dryness of the nasal mucosa can be an additional burden for rhinosinusitis patients and result in pain and nose bleeding.

The effectiveness of treatment with Ectoin^®^ Rhinitis Spray was also reflected in rhinoscopic results as the number of patients without the presence of endonasal oedema and endonasal redness was clearly higher in the groups using Ectoin^®^ Rhinitis Spray (either as monotherapy or as concomitant therapy).

The evaluation of the general health status over time by the treating physician showed no statistical significant differences between the three treatments although at visit 2 more patients in the ectoine group and in the ectoine + Xylo group were in good health compared to the Xylo group (58.9 and 53.6%, respectively vs 42.9%).

Clear improvement of all symptoms was also reflected in the patient diaries of all three treatment groups upon treatment. The usage of a diary based on the validated disease-specific SNOT-20 questionnaire allowed a measure both of health and of quality of life aspects. Comparing mean values of single symptoms showed some significant differences between groups on single days. However, percentual decreases of symptoms over the entire study duration were comparable between groups except the parameters ‘dry nose’ (better improvement in the ectoine group compared to the Xylo group), ‘ear pain’ (better improvement in the combination group compared to the ectoine group) and the symptom ‘fatigue’ (better improvement in the Xylo group compared to the ectoine group).

The judgment of the overall efficacy of both products demonstrated that the efficacy was judged favourable for Xylometazoline compared to ectoine by physicians and by patients. Importantly, the judgment of the tolerability demonstrated a significantly better tolerability of the Ectoin^®^ Rhinitis Spray compared to Xylometazoline (both applied as monotherapy or as combination therapy) as assessed by physicians and by patients. Thus, it was demonstrated that the Ectoin^®^ Rhinitis Nasal Spray was very well tolerated both if applied with or without concomitant application of Xylometazoline.

The analysis of Xylometazoline doses demonstrated that the applied doses were significantly lower in the combination group on days 1 and 3 compared to the group applying Xylometazoline alone. This may indicate that the concomitant application of Ectoin^®^ Rhinitis Nasal Spray had a positive influence on the used amount of medication, allowing the patients to reduce the doses of Xylometazoline.

Of note, results showed that allowed daily doses of 3 Xylometazoline sprays were exceeded by many patients, indicating that the patients did not adhere to the limitations given in the instructions for use. This may suggest that patients require higher doses of Xylometazoline for adequate reduction of symptoms as foreseen for this product. As no dosage limitations are specified for Ectoin^®^ Rhinitis Nasal Spray, it could be administered on an as-needed basis without dosage restrictions. Importantly, the overuse of decongestant nasal sprays has been linked to rebound congestion [22], a risk which can be ruled out for the Ectoin^®^ containing nasal spray which works solely on a physical mode of action [9]. Importantly, the biological safety evaluation of the Ectoin^®^ Rhinitis Spray showed that there is no safety concern regarding the ingredients of this formulation, even if used for long-term periods. A study with chronic rhinosinusitis patients applying the Ectoin^®^ Rhinitis Spray for a longer period as in the current study would be very interesting. Of note, application of Ectoin^®^ containing eye drops over a period of 6 months has already been reported [23] as well as 3 months intranasal application in children [24].

Patients expressed their satisfaction with Ectoin^®^ Rhinitis Spray treatment as 67.9% of patients stated that they would use the nasal spray again. This is particularly true for the combination treatment: 78.6% of patients stated that they would use the combination Ectoin^®^ Rhinitis Spray and Xylometazoline again.

The findings from the current study are in line with results from another study, where Ectoin^®^ Rhinitis Spray was compared to phytotherapeutic tablets (Sinupret forte) in patients with acute rhinosinusitis. As similar outcome measurements were chosen as in the current study, results can be compared to the former trial, which demonstrated that the Ectoin^®^ nasal spray was as effective as the phytotherapeutic tablets in treating acute rhinosinusitis [13].

Findings from the current study are also supported by results of clinical trials with allergic rhinitis patients, investigating very similar symptoms as in acute rhinosinusitis. Comparisons of efficacy and safety of an Ectoin^®^ containing allergy nasal spray with a glucocorticoid nasal spray, an antihistamine nasal spray and a cromoglicate acid nasal spray confirmed the effective symptom reduction and a very good safety profile of the Ectoin^®^ containing product [12, 25]. In addition, treatment of rhinitis sicca with Ectoin^®^ containing nasal sprays demonstrated clinical and statistically significant improvement of nasal airway obstruction and crust formation and very good tolerability [26].

After completion of the study, the EPOS 2020 guideline was released. Interestingly the diagnosis of ARS has not substantially changed, but the guideline has been updated with current clinical data. There is still a need for effective treatment options for acute rhinosinusitis. Thus, according to the EPOS guideline 2020, symptomatic treatment of common cold with nasal corticosteroids or (mid to long term) treatment with antihistamines is currently not recommended, and the application of paracetamol has been shown to improve nasal obstruction and rhinorrhoea, whereas other symptoms of the disease stay unaffected. Similarly, non-steroidal anti-inflammatory drugs (NSAIDs) are recommended for relieving discomfort or pain caused by the common cold without improving other symptoms. Combination preparations of antihistamines, analgesics and decongestants were shown to be beneficial in adults and adolescents but not in young children, but risk–benefit evaluation should be performed regarding potential side effects [21]. Nasal saline irrigation demonstrated benefits for relieving symptoms of acute rhinosinusitis, and a significant reduction in the concomitant use of decongestants by a saline group was observed [27]. Of note, a comparison of the EPOS 2012 and 2020 versions indicates that the function of the nasal epithelium as a barrier against invading respiratory viruses has gained increasing importance. This goes in line with the results of the current study where the membrane-stabilizing molecule Ectoin^®^ was used, thereby supporting the use of Ectoin^®^ containing Rhinitis Spray as a valuable treatment option of acute rhinosinusitis, both, as stand-alone or in combination with decongestion nasal sprays.

The design as an observational study might be a potential weakness of the current study. Following German regulations, CE-certified medical devices, if applied within their intended use and without invasive or burdensome measurements may be investigated in accordance with § 23b of the German Medical Device Act (MPG). Comparably, drugs may be investigated in drug observational studies (AWB) if they are applied in accordance with their summary of product characteristics and following current medical practice. A consequence of choosing this study design in accordance with the German Medicinal Products Act (AMG) is the fact that both, randomization of patients and the inclusion of a placebo group is not allowed. Thus, although it is accepted that randomization may minimize selection bias as treatment group assignment is performed by chance and that treatment groups may be balanced better regarding unknown confounding variables, it is not always a realisable methodology. Similarly, the use of placebo treatment (being defined as treatment designed to have no therapeutic effect), has been discussed contradictory. Thus, it has been demonstrated that double-blind randomized placebo-controlled trials also have their limitations and disadvantages, and that patients’ awareness of a placebo arm can lead to modifications of results due to patients’ expectations and interpretations [28, 29]. Of note, a placebo treatment in the current study would have been realisable only as a saline nasal spray (without the key ingredient Ectoin^®^ or the decongesting compound Xylometazoline). As it is known that saline nasal sprays do have positive effects on ARS symptoms, they would not have been suitable as placebo treatment here. It should be pointed out that an observational study design is increasingly accepted as a valuable source of clinical evidence [30, 31]. Although observational studies might have disadvantages in comparison to randomised controlled trials (RCTs), they might on the other hand be more suitable in terms of reflecting what is achieved in standard medical practice. A critical point to be considered in observational studies is the allocation of patients to one or another treatment or control group which it not random and can therefore depend on subjective measurements. To keep this risk as low as possible, the current study had clearly defined in- and exclusion criteria and patients had to show a certain degree of symptoms at inclusion (reflected e.g., by a minimum of total nasal symptom score values) to ensure homogeneity of patients. Further, the application of a validated patient questionnaire as well as statistical analysis techniques ensured the achievement of scientifically valid study data. Importantly, sites specialized in ear nose throat practice were chosen to warrant a very precise assessment of symptoms by specialized physicians.

## Conclusion

It can be concluded that acute viral rhinosinusitis can be successfully treated with Ectoin^®^ Rhinitis Nasal Spray, resulting in effective reduction of rhinosinusitis symptoms and improvement of concomitant physical and emotional complaints. Overall, the study demonstrated that the level of reduction of rhinosinusitis symptoms was comparable to that achieved with the pharmacologically acting Xylometazoline nasal spray, which is accepted as standard care treatment of rhinosinusitis. Importantly, one of the hallmark negative effects of Xylometazoline, the disturbance of the nasal epithelial barrier, reflected by the symptom of nasal dryness does not develop when applying Ectoin^®^ Rhinitis Nasal Spray. Concomitant treatment with both products demonstrated that the development of dry nasal mucosa could even be counteracted. Simultaneously, the presence of endonasal oedema and endonasal redness can be diminished using Ectoin^®^ Rhinitis Nasal Spray (either as monotherapy or as concomitant therapy). In line with this, the overall tolerability of Xylometazoline treatment was judged better when used in combination with Ectoin^®^ Rhinitis Spray, thus supporting its use as a concomitant application. It would be desirable to substantiate the current results with additional data from a randomised controlled study in the future.

Taken together, treatment with Ectoin^®^ Rhinitis Spray alone or together with Xylometazoline can be recommended as treatment of acute rhinosinusitis and represents a simple and inexpensive therapy with a very good safety profile.

## Data Availability

The datasets generated during and/or analysed during the current study are available from the corresponding author on reasonable request.
